# Effectiveness of Vitamin C for Gingival Depigmentation: A Systematic Review

**DOI:** 10.1155/ijod/6980625

**Published:** 2026-01-20

**Authors:** Bushra Aljahdali, Rana Dahlan

**Affiliations:** ^1^ Department of Periodontology, Faculty of Dentistry, King Abdulaziz University, Jeddah, Saudi Arabia, kau.edu.sa; ^2^ Department of Public Health Dentistry, Faculty of Dentistry, King Abdulaziz University, Jeddah, Saudi Arabia, kau.edu.sa

**Keywords:** ascorbic acid, depigmentation, gingival depigmentation, gingival hyperpigmentation, vitamin C

## Abstract

**Objectives:**

This systematic review is meant to evaluate the effectiveness of ascorbic acid (vitamin C) in gingival depigmentation and assess its potential as a minimally invasive alternative for managing gingival hyperpigmentation.

**Materials and Methods:**

Electronic searches were conducted across major databases, yielding eight relevant studies, including five randomized clinical trials and three case series. These studies reported different application techniques, with intramucosal injection (mesotherapy) being the most frequently used approach.

**Results:**

All studies demonstrated improvement in gingival pigmentation following vitamin C therapy, as assessed by indices such as the Dummett‐Gupta oral pigmentation index (DOPI). Mesotherapy generally produced faster and more pronounced results than topical gel application. High patient satisfaction and minimal adverse effects were consistently reported. Nevertheless, marked heterogeneity in study design, dosing, and outcome evaluation limited direct comparison among studies.

**Conclusions:**

Vitamin C shows promise as an effective, safe, and minimally invasive treatment for gingival depigmentation. However, standardized clinical protocols and long‐term randomized trials are required to establish its comparative efficacy and ensure consistent outcomes.

## 1. Introduction

Gingival esthetics is a critical aspect of dental health and appearance that influences both professional assessment and patient satisfaction [1]. Unsightly gum discoloration, often referred to as dark gingiva, is a common esthetic concern among patients. The visibility of dark pigmentation while smiling or speaking can negatively affect self‐confidence and perceived oral attractiveness, prompting many individuals to seek cosmetic correction [2, 3]. Gingival pigmentation typically results from melanin deposition in the basal and suprabasal epithelial layers and may be physiological or acquired, depending on genetic, environmental, or systemic factors [2–4]. Internal causes include genetic disorders, hormonal imbalances (such as Addison’s disease, Albright and Nelson’s syndrome, and acromegaly), inflammatory processes, and both benign and malignant growths [3]. Dark gingiva may also stem from certain medical conditions, including Kaposi’s sarcoma, malignant melanoma, Peutz‐Jeghers syndrome, injury, chronic lung disease, and hemochromatosis [2, 3]. External causes include discoloration from heavy metal consumption/toxicity, amalgam tattoos, tobacco use, and extended use of certain medications, such as antimalarial drugs, tricyclic antidepressants, and birth control pills [5–7]. Physiological pigmentation is common in many populations and represents a normal variation in melanin expression [4, 8–10]. However, environmental and lifestyle factors, particularly smoking, can intensify existing pigmentation or trigger new melanin deposition in the gingiva [4, 10]. The multifactorial nature of this condition highlights the importance of distinguishing physiological from acquired pigmentation when assessing patients and planning esthetic interventions [4, 8, 9, 11].

Various indices have been developed to assess the degree and severity of gingival pigmentation [12]. One of the most frequently used is the Dummett‐Gupta oral pigmentation index (DOPI), which provides a numerical score to represent the level of melanin pigmentation seen during the clinical assessment of different oral tissues [4].

The literature reports a wide range of gingival depigmentation techniques, which can be broadly classified into two main categories. Surgical approaches include gingival abrasion, split‐thickness epithelial excision, free gingival grafting, electrosurgery, and various laser modalities such as CO_2_, diode, Nd:YAG, Er:YAG, and argon lasers [13–15]. Chemical approaches involve the use of agents such as alcohols, phenols, and ascorbic acid [15]. While various treatment approaches have been explored, vitamin C has recently gained attention as a promising, minimally invasive, nonsurgical alternative for gingival depigmentation [15, 16].

Ascorbic acid (vitamin C) demonstrates the ability to suppress tyrosinase, an enzyme that is critical for melanin biosynthesis [17]. This inhibitory action leads to decreased melanin production, establishing vitamin C as an effective depigmenting agent [16, 18]. The ability of vitamin C to mitigate dark skin pigmentation is primarily attributed to its antioxidant properties and role in impeding melanin synthesis [19, 20]. Vitamin C acts as a potent antioxidant that protects the skin against oxidative stress induced by ultraviolet (UV) radiation, which can trigger increased melanin production and pigmentation. In addition to suppressing melanin synthesis by interfering with tyrosinase activity, vitamin C impedes melanin formation; this interference results in reduced pigmentation and contributes to the skin‐lightening effects of vitamin C [21, 22]. Vitamin C has been widely used to lighten darkened skin areas through topical, transdermal, or systemic delivery routes, and this water‐soluble antioxidant is crucial for cellular function [12, 23]. Despite its vital importance, however, humans lack the ability to produce this vitamin internally due to a genetic mutation affecting its synthesis pathway [24].

Vitamin C is used in various gingival depigmentation methods, and some such techniques include oral mesotherapy (intramucosal injection), in which vitamin C is injected directly into the gingiva [16]. This method has shown a significant reduction in pigmentation indices and high patient satisfaction, owing to its minimally invasive nature. Patients typically receive injections once per week for several weeks, with noticeable depigmentation effects observed within a month [16]. Vitamin C can also be topically applied as a gel or cream directly to the gingiva [25]. This method is less invasive but may take longer to show results compared to injections. Topical application is often used in conjunction with microneedling, which enhances the penetration of vitamin C into the gingival tissues. Microneedling with topical vitamin C involves the creation of micro holes in the gingiva using Dermapen, followed by the application of vitamin C. It is a nonsurgical and cost‐effective method that has shown promising esthetic results. After surgical depigmentation procedures, vitamin C is sometimes applied to aid in healing and maintain the depigmentation effect. It has been shown to improve healing and reduce postsurgical pain [26].

This systematic review is intended to compile current evidence on the effectiveness, safety, and long‐term results of ascorbic acid use for gingival depigmentation.

## 2. Materials and Methods

### 2.1. Protocol and Registration

The protocol for this systematic review has been registered with PROSPERO under the registration number CRD42025646779 (http://www.crd.york.ac.uk/prospero/). The review was carried out and documented following the guidelines of the Cochrane Handbook [27] and the Preferred Reporting Items for Systematic Reviews and Meta‐Analyses (PRISMA) statements for systematic reviews in health sciences [28].

### 2.2. Eligibility Criteria

Based on the Participants–Intervention–Comparison–Outcome–Study (PICOS) method, we included human clinical studies of various designs published in English that reported before‐and‐after outcomes of local application of vitamin C for gingival hyperpigmentation. The study population included adults with gingival hyperpigmentation. The intervention involved application of topical vitamin C. A comparison was made between the baseline and postapplication assessments to evaluate changes in the degree of gingival melanin pigmentation. The outcome measures included the degree of gingival depigmentation, patient satisfaction, and the percentage of regimentation. There were no restrictions on age, follow‐up duration, or years of publication. Review articles, letters to the editor, personal opinions, animal studies, and articles not published in English were excluded from the literature review.

### 2.3. Data Sources and Search Strategy

A comprehensive search was performed up to January 2025, using the PubMed/MEDLINE, Web of Science, and Scopus electronic bibliographic databases. A search strategy was developed, with the search terms defined in PubMed and subsequently adapted for use in various electronic databases. The following search terms were used to search all databases: “vitamin C,” “ascorbic acid,” “melanin production,” “gingival hyperpigmentation,” “melanocytic activity,” “depigmentation,” “adults,” “patients,” and “humans.” Manual screening was completed by examining bibliographies and reference lists from the selected papers to identify any potential studies that might have been missed during the electronic search. In the final step, a search for gray literature was performed using Google Scholar and the Google search engine.

### 2.4. Study Selection

Each reviewer independently examined the titles and abstracts to find papers that might be relevant according to the inclusion criteria. If the abstracts lacked sufficient details, the full articles were assessed to decide if they met the selection criteria. In cases where there was a disagreement in the selection process, the reviewers discussed the matter until they reached an agreement.

### 2.5. Data Extraction and Data Items

Data from the chosen papers were independently extracted by two reviewers, focusing on the following items: year of publication, country, type of study, sample size, mean age of participants, sex of participants, inclusion and exclusion criteria, gingival pigmentation index (GPI) used, phase one therapy performed, methods of gingival depigmentation, vitamin C concentration, follow‐up intervals, statistical analysis used, main findings, postoperative complications, pigmentation index, methods of evaluation of patient satisfaction, and patient satisfaction. Inconsistencies between each author’s findings have been discussed and resolved. Information that was either missing or unclear was acquired from the authors of the selected papers.

### 2.6. Risk of Bias in Individual Studies

Each reviewer conducted independent evaluations of the methodological quality, following the PRISMA guidelines [28]. The Joanna Briggs Institute (JBI) critical appraisal was used for the case series [29], and Version 2 of the Cochrane risk of bias tool for randomized trials (RoB 2) [30].

## 3. Results

### 3.1. Search Results

**Figure 1:**
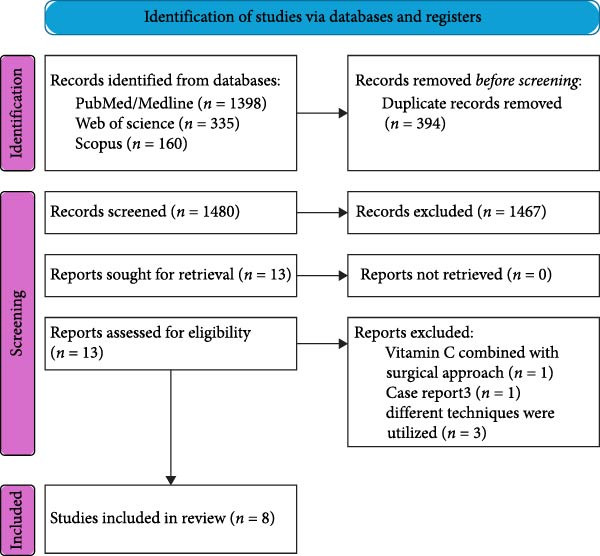
PRISMA flowchart for search details and excluded studies.

Following the electronic search, 1893 articles were found, and 394 duplicates were excluded, leaving 1480 articles. After title and abstract screening, 1467 were excluded because they did not meet the eligibility requirements. Full‐text screening was performed on 13 articles. Eight studies were included. Details of the literature search and study selection are demonstrated in Figure 1 and Table 1.

**Table 1:** Characteristics of excluded studies.

Study	Reason for exclusuion
Mostafa D, Alotaibi SM. A successful esthetic approach of gingival depigmentation using microneedling technique and ascorbic acid (Vitamin C). *Case Rep Dent*. 2022;2022:3655543. published 2022 Apr 25	Case report
Sandhu A, Jyoti D, Sharma H, Phull T, Khurana NS, Tiwana JK. Efficacy of topical vitamin c application on healing after gingival depigmentation by scalpel: a case series. *Cureus*. 2023;15(11):e48417. published 2023 Nov 6	Ascorbic acid was combined with surgical procedure
Shah, Rahul & Thomas, Biju & Madani, Mohamed & Shetty, Shamila. (2013). Gingival depigmentation: case series for four different techniques. Journal of health and allied sciences NU. 03. 10.1055/s‐0040‐1703720.	Utilized different techniques for gingival depigmentation
Thangavelu A, Elavarasu S, Jayapalan P. Pink esthetics in periodontics ‐ Gingival depigmentation: a case series. *J Pharm Bioallied Sci*. 2012;4(Suppl 2):S186‐S190	Utilized different techniques for gingival depigmentation
Kasagani SK, Nutalapati R, Mutthineni RB. Esthetic depigmentation of anterior gingiva. A case series. *N Y State Dent J*. 2012;78(3):26‐31.	Utilized different techniques for gingival depigmentation

### 3.2. Characteristics of the Included Studies

Of the eight studies that were included, five were randomized clinical trials [8, 16, 25, 31, 32], and three were case series studies [9, 11, 33]. Table 2 offers a comprehensive overview of the studies that have been included.

**Table 2:** Characteristics of included studies.

Author, year	Country	Type of study	Sample size	Mean age of participants	Gender	Inclusion criteria	Exclusion criteria
Mostafa et al. (2023) [33]	Saudi Arabia	Case series	16	24.8 ± 4.73 years	50% males (*n* = 8) 50% females (*n* = 8)	Health‐focused individuals aged 14 to 50 who have not undergone any prior treatments impacting their gum health or color. These individuals exhibit mild‐to‐severe melanin pigmentation in the anterior gums, as classified by Dummet	Edentulous patients, women who are pregnant or breastfeeding, individuals who smoke, and those on medications affecting gum color or with systemic diseases that may trigger melanin production
Esmat et al. (2023) [32]	Egypt	Randomized clinical trial	26	23 ± 2.31 years	15.4% males (*n* = 2) 84.6% females (*n* = 11)	Individuals aged 18 to 40, regardless of gender, who have hyperpigmentation in the front esthetic area of the upper or lower gingiva and are not affected by any systemic illnesses or conditions	Individuals who smoke, women who are pregnant or breastfeeding, those taking medications linked to gingival melanin pigmentation such as antimalarial drugs, phenothiazines, and oral contraceptives, as well as patients with periodontal disease or known sensitivity to ascorbic acid
Dawar et al. (2022) [11]	India	Case series	6	Not reported	Not reported	Presence of physiological melanin hyperpigmentation in the front area of the upper or lower jaw, commitment to attend weekly sessions for 4 to 5 weeks in a row, being over 18 years old, maintaining good oral hygiene, and absence of any systemic diseases	Individuals who smoke, women who are pregnant or breastfeeding, those with gingival pigmentation caused by disease or medication, patients using vitamin C supplements for other purposes, and those with a known allergy to vitamin C
Swarna and Subhasree (2024) [25]	India	Randomized clinical trial	16	26.7 ± 5.67 years	Not reported	Individuals ranging in age from 18 to 35 years, showed overall good health, and had physiological gingival hyperpigmentation in the esthetic area	Individuals with bleeding conditions, those receiving chemotherapy, expectant mothers, tobacco users, and people with gingivitis or periodontitis
Ul Haque et al. (2024) [31]	India	Randomized clinical trial	21	29.8 years	38% male 61.9% females	Individuals aged 18 and older, those experiencing hyperpigmentation in anterior gingiva, patients open to receiving treatment for gingival hyperpigmentation, and those who are systemically healthy	Individuals with a history of smoking or tobacco use, women who are pregnant or breastfeeding, those with gingival thickness of 1.5 mm or less, individuals taking medications that might influence gingival pigmentation, those with systemic conditions that could lead to gingival hyperpigmentation, and patients conditions that might affect gingival color
Chaudhary et al. (2023) [8]	India	Randomized clinical trial	15	18–40 year	60% females and 40% males	Individuals who are systemically healthy, with a Dummett and Bolden index score of 1, 2, or 3, who maintain good oral hygiene, have esthetic concerns, and are open to undergoing minor surgical procedures	Individuals with systemic conditions linked to abnormal hyperpigmentation or delayed wound healing, such as uncontrolled diabetes and autoimmune disorders, as well as those with untreated periodontal disease, gum recession, fenestration and dehiscence, moderate to severe gingivitis, chronic smokers, and those who do not adhere to treatment plans
El‐Mofty et al. (2021) [16]	Egypt	Randomized clinical trial	20	Group I was 27.3 ± 6.5 years Group II was 27.2 ± 6.8 years	20% male 80% female	Individuals classified as ASA (American Society of Anesthesiology) Type 1 with melanin pigmentation in the front section of either the upper or lower gingiva	Individuals who smoke, are pregnant or breastfeeding, have mental retardation, suffer from gum disease, possess nonvital front teeth, have active tooth decay, are allergic to vitamin C, or have undergone previous skin depigmentation treatments
Lavanya et al. (2024) [9]	India	Case series	3	22–23 years	Not reported	Not reported	Not reported

Most studies involved the use of ascorbic acid via intramucosal injection [8, 11, 16], and two studies applied microneedling using a Dermapen device and then applied vitamin C powder topically [25, 33]. One study compared the intramucosal injection technique with the topical application of vitamin C gel [16], one study compared vitamin C application to diode laser usage [32], and three studies compared vitamin C to surgical depigmentation using a scalpel [8, 25, 31]. The various studies used different ascorbic acid application techniques, the majority of which implicated the mesotherapy technique, also known as intramucosal injection, in which an insulin administration syringe with a 30‐gauge needle was inserted at 0.5–2 mm and ~0.1 mL vitamin C solution was injected, and the process was repeated across the pigmented area at 2–3 mm distance [8, 9, 11, 16, 31, 32]. Two studies used the microneedling technique using a Dermapen device, which features adjustable penetration depth settings to accommodate a range between 0.2 and 3 mm, depending on the gingiva’s thickness. Once the bleeding sites were identified, a mixture of topical ascorbic acid powder (1000 mg/mL) and saline was applied to the gingival tissue for 10 min [25, 33]. In one study, patients were directed to use a gel containing 10% ascorbic acid, propylene glycol, and hydroxypropyl methylcellulose (HPMC) once a day for a duration of 3 months [16].

Gingival color was evaluated, and the initial pigmentation score was recorded using DOPI [4] in seven studies [8, 9, 11, 16, 25, 32, 33]. The Hedin melanin index (HMI) was also used in one study [33], the GPI in three studies [11, 31, 32], and the skin hyperpigmentation index (SHI) in one study [31]. Nonsurgical periodontal therapy was described in six studies prior to the depigmentation procedure, which mainly consisted of professional scaling, patient motivation, and oral hygiene instruction [8, 9, 11, 16, 32, 33].

The depigmentation procedure was repeated after 2 weeks in one study [33] and weekly for 4 weeks in another five studies [8, 9, 11, 31, 32]; two studies mentioned that patients received three treatment sessions at 1‐week intervals [16, 25]. Postoperative instructions included not consuming spicy or acidic food and avoiding brushing of the treated area for 1 to 3 days.

### 3.3. Effect of Ascorbic Acid on Gingival Depigmentation

DOPI and GPI scores were the main parameters used to evaluate the degree of gingival depigmentation, with a follow‐up period ranging from 2 weeks to 6 months and a final follow‐up time ranging from 1 month to 2 years. Additional details on vitamin C application are provided in Table 3.

**Table 3:** Details of vitamin C application and study findings.

Author, year	Gingival pigmentation index used	Initial periodontal treatment	Method of gingival depigmentation	Follow‐up intervals	Statistical analysis	Main findings
Mostafa et al. (2023) [33]	Dummett‐Gupta oral pigmentation index (DOPI): to assess pigmentation intensity scores (PIS) The Hedin melanin index (HMI): to assess pigmentation extension scores (PES)	All participants received professional teeth cleaning and oral hygiene instructions before treatment	In a completely sterile environment and under infiltration anesthesia, microneedling was performed on the anterior pigmented gingiva using a Dermapen with 1.5 mm deep needles. Once bleeding points were visible on all treated gingiva, a mixture of topical ascorbic acid powder (1000 mg/mL) and saline was applied to the gingival mucosa for 10 min. The treated area was left uncovered. The same microneedling and ascorbic acid application procedure was repeated after 2 weeks	Three distinct time points: prior to treatment (baseline), 2 weeks following treatment, and 1 month posttreatment	Paired *T*‐tests	The average DOPI score showed a significant reduction, dropping from 3.31 ± 0.60 at the start to 1.44 ± 0.51 after 1 month (mean difference: 1.8 ± 0.7, 95% CI: 0.17–1.49, *p* ≤ 0.001). In a similar pattern, the mean HMI score decreased from 3.81 ± 0.40 to 0.69 ± 0.70 (mean difference: 3.1 ± 0.7, 95% CI: 2.74–3.50, *p* ≤ 0.001). All patients achieved outstanding esthetic outcomes, with seven experiencing complete gingival depigmentation and nine showing a reduction in their indices
Esmat et al. (2023) [32]	Dummett‐Gupta oral pigmentation index (DOPI): used to evaluate the intensity of pigmentation in the gingiva. Gingival pigmentation index (GPI): employed to determine the extent or pattern of pigmentation in the gingiva	Participants received professional scaling and oral hygiene instructions	Patients were prepared to ensure their oral health was in optimal condition. Infiltration anesthesia was applied to the designated area. Vitamin C was administered through intramucosal injections (mesotherapy) using 1–1.5 mL of Cevarol (L‐ascorbic acid 1000 mg/5 mL, Memphis Pharmaceuticals and Chemical Industries, Cairo, Egypt). An insulin syringe (30 gauge 1 cc 0.33 mm × 8 mm 5/16″ needle) was used for the injections. The needle was inserted just below the gingival epithelium’s surface, positioned parallel to the junction of the epithelium and connective tissue. The needle’s bevel was oriented upward, and 0.1 mL was injected at each site until the tissue blanched. This procedure was repeated, maintaining a 2 to 3 mm gap between injection points. Each patient received four treatment sessions, with a 1‐week gap between each session	One month, 2 months, and 3 months after the treatment procedure was completed	The Friedman test was utilized to evaluate changes over different time periods, followed by a post hoc test with Bonferroni correction. Pearson’s Chi‐square and Fisher’s exact tests were used to assess the differences in patient satisfaction	The mean DOPI score significantly decreased from 2.46 ± 0.42 at baseline to 0.95 ± 0.35 after 3 months (*p* = 0.043), with a significant reduction observed at 1, 2, and 3 months. Similarly, the mean GPI score declined from 2.54 ± 0.52 to 1.92 ± 0.64 (*p* < 0.0001) across different time intervals
Dawar et al. (2022) [11]	Dummett‐Gupta oral pigmentation index (DOPI) measures pigmentation intensity, while the gingival pigmentation index (GPI) assesses the extent of pigmentation. Digital photographs are analyzed using digital image analysis. The pigmented surface area (PSA) is determined by tracing the pigmented region with the software’s quick selection tool, and the area is measured. If there are isolated pigmentation patches, each is selected separately to calculate the total pigmented gingiva area. Gingival luminescence (*L* ^∗^) or lightness is evaluated using the CEILAB system (International Commission on Illumination *L* ^∗^ *a* ^∗^ *b* ^∗^ color order system), where a specific shade is defined by the coordinates *L* ^∗^, *a* ^∗^, and *b* ^∗^, representing human color perception. *L* ^∗^ is the vertical axis indicating lightness, ranging from 0 to 100, with 100 being the lightest. 15, 16 The *L* ^∗^ value for gingiva around each tooth is recorded using software to compute the mean value for the entire pigmented region S100 stains. Photomicrographs are taken with a digital camera at 20× magnification	Full‐mouth sprofessional scaling	Following infiltration anesthesia. An intraepidermal gingival injection, using the oral mesotherapy technique, was administered with 1.5–2.0 mL of L‐ascorbic acid at a concentration of 250 mg (V–C injection, 2 mL ampoule containing 500 mg ascorbic acid, Vesco Pharmaceutical). This procedure utilized a 30‐gauge insulin syringe, with the needle penetrating to a depth of 0.5 to 2.0 mm. Approximately 0.1 mL of vitamin C was injected locally until the gingiva blanched at each point, spaced 2–3 mm apart. This process was repeated weekly for four sessions, except for one patient who required a fifth visit	1 and 3 months after the injection appointments	Due to the limited sample size (*n* = 5), nonparametric tests that utilize the median were selected instead of the mean. The Friedman test and the Wilcoxon signed‐rank test were employed to evaluate changes in the parameter over time and across various time points	Initially, all five patients exhibited moderate to severe gingival pigmentation, with baseline median scores for GPI and DOPI recorded at 3.0 and 2.3, respectively. By the third month, these scores had notably reduced to 1 and 0.8. A statistically significant change was noted in the median values of DOPI, GPI, *L* ^∗^, and PSA when comparing baseline data to 1‐month data using clinical and photographic assessments. Furthermore, a significant change in the *L* ^∗^ value was observed between the first and third months. Histological examination showed a significant difference in MHC from baseline to 3 months
Swarna and Subhasree (2024) [25]	Dummett‐Gupta oral pigmentation index (DOPI)	Not reported	Following local anesthesia, microneedling was performed using a Dermapen device (Dr. Pen, Las Vegas, NV), and a topical mixture of ascorbic acid powder (1000 mg/mL) and saline was applied to the gingiva for a duration of 10 min. The device features an adjustable penetration depth, which can be set between 0.2 and 3 mm depending on the thickness of the gingiva, using the device’s settings. It also offers six‐speed modes, ranging from 412 cycles per minute at the lowest speed to 700 cycles per minute at the highest speed. Patients received three treatment sessions at 10 days interval	Assessments of the scores were conducted initially, then again after 1 month, and finally 3 months following the depigmentation treatments. Measurements of wound healing were taken on both the first and seventh days after surgery	The mean and standard deviations of the scores were calculated. A one‐way ANOVA test was conducted for DOPI to compare various time intervals (baseline, first month, and third month) within the same group	The mean DOPI score significantly decreased from 2.61 ± 0.17 at baseline to 0.87 ± 0.17 by the end of the third month. A statistically significant change in median DOPI was found between the baseline and 1 month (*p* < 0.001 ^∗^), as well as between the baseline and 3 months (*p* < 0.001 ^∗^)
Ul Haque et al. (2024) [31]	Gingival pigmentation index (GPI) and skin hyperpigmentation index (SHI)	Not reported	Initially, 15% lignocaine was applied as a topical anesthetic, with one spray per quadrant. After drying the area, a vitamin C solution was injected through several pricks. Using an insulin syringe equipped with a 30‐gauge needle, 1.25 mL of 10% vitamin C was administered. With the needle aligned parallel to the gingival surface, around 0.1 mL of the solution was administered at each injection site. After each treatment session, patients were instructed to take oral pain killers (aceclofenac 15 mg + paracetamol 325 mg) twice a day for the first 24 h following the procedure and as needed afterward. This process was repeated weekly, up to a maximum of four sessions, or until no further color change was noted, whichever occurred first	Baseline, 1 and 6 months	The analysis of results was conducted using descriptive statistics and by comparing the study groups. The SHI was compared using an unpaired *t*‐test. The datasets for GPI and VAS scores were evaluated with the Mann–Whitney test	During the 1‐month postoperative check‐up, the GPI score dropped from an initial 2.24 to 0.29, although there was no notable difference between groups (Mann–Whitney test, *p* = 0.541). At the 6‐month mark, the GPI score rose slightly to 0.67 but remained similar (Mann–Whitney test, *p* = 0.211). Likewise, SHI scores fell from 1.55 at the start to 1.05 after 1 month and 1.11 after 6 months, reflecting a consistent outcome over the period
Chaudhary et al. (2023) [8]	Dummett‐Gupta oral pigmentation index (DOPI)	Full mouth supragingival and subgingival scaling and oral hygiene instructions	Following topical anesthesia, 2 mL vitamin C ampule was injected into the gingiva at the epithelial connective tissue interface until the tissue turned white, with 0.1–0.2 mL (200–300 mg vitamin C) administered every 2–3 mm apart, utilizing an insulin syringe (needle–30 gauge and 8 mm in length). The syringe was inserted into the gingival tissues with the bevel pointing upward. The vitamin C was given once every week over a period of 4 weeks	Depigmentation scores were evaluated after 1 week. Repigmentation was evaluated at 3 months	Descriptive statistics are presented as mean ± standard deviation (SD). An independent *t*‐test was conducted to compare the pigmentation area, while the Mann–Whitney *U* test was employed to assess differences in pigmentation intensity, repigmentation, and VAS score between the groups	Mean preoperative GPI score was 114.07 ± 13.24 and reduced to 37.07 ± 5.99 1 week postoperatively
El‐Mofty et al. (2021) [16]	Dummett‐Gupta oral pigmentation index (DOPI)	Professional scaling and oral hygiene instructions	Group 1 received an intramucosal injection of vitamin C: The area was anesthesized using a field block technique, followed by intramucosal injections of 1 mL Cevarol (L‐ascorbic acid 1 mg/5) administered locally. A (29 gauge 1 cc 0.33 mm x 8 mm 5/16″ needle) was used, ensuring that no more than 1 mm of the needle was inserted beneath the epithelial surface, parallel to the epithelium‐connective tissue junction across the pigmented area until the tissues blanched (0.1 mL per point, spaced 2–3 mm apart). Each patient underwent this treatment three times, with a 1‐week gap between sessions, with all injections performed by the same operator. Group 2: vitamin C topical gel: Patients were instructed to apply a gel containing 10% ascorbic acid, along with propylene glycol and hydroxypropyl methylcellulose (HPMC), once daily for 3 months	2‐week recall appointments	To study the changes in DOPI within each group, Friedman’s test was employed. For pairwise comparisons, Dunn’s test was utilized. The Wilcoxon signed‐rank test was applied to assess differences in area fractions between the baseline and after 6 months. Qualitative data were expressed as frequencies and percentages. Fisher’s exact test was conducted to compare the groups. The threshold for significance was established at *p* ≤ 0.05	Throughout all follow‐up periods, the study groups showed no significant diffirence in their median DOPI. Nonetheless, over time, Group 1 showed a statistically significant alteration in DOPI, with a *p* value of less than 0.001 and an effect size of 0.9. Analyses over various time periods revealed a statistically significant decrease in the median DOPI during the initial month. Conversely, Group 2 exhibited no statistically significant variation in DOPI over time (*p* value = 0.223, effect size = 0.146)
Lavanya et al. (2024) [9]	Dummett‐Gupta oral pigmentation index (DOPI)	Full mouth oral prophylaxis and oral hygiene instructions were given	After topical anesthesia was applied, an intraepidermal gingival injection, utilizing the oral mesotherapy technique, was performed. For precise drug delivery, a syringe employed for insulin administration with a 30‐gauge needle was used. The needle was inserted at a depth of 0.5–2.0 mm. 1.5–2.0 mL of vitamin C with a concentration of 250 mg (0.1 mL) was approximately administered locally at points spaced 2–3 mm apart until gingival blanching occurred. The same procedure was repeated once in 4 weeks	One‐, 3‐ and 6‐month intervals	NA	DOPI and GPI showed significant improvement at 1 month when compared to the initial baseline. A gradual reduction in pigmentation and an enhanced pinkish color were noted during the 1‐, 3‐, and 6‐month follow‐ups. Gingival color did not significantly change beyond 1 month, indicating no further repigmentation post the 1‐month follow‐up

*Note: L* ^∗^, Luminescence value. *a* ^∗^ and *b* ^∗^, indicates sample chromaticity; *a* ^∗^ (red/green axis) and *b* ^∗^ (yellow/blue axis).

All included studies demonstrated favorable outcomes after vitamin C administration regardless of which of the diverse application methods was employed. Vitamin C mesotherapy was compared with vitamin C topical gel application in one study, and the mesotherapy technique revealed a better and quicker effect than the topical gel [16].

Microscopic examination of tissue samples was performed in one study, which indicated a notable variation in the melanocyte histopathologic count (MHC) between the initial measurement and the 3‐month mark [11]. Postoperative repigmentation was reported in one study, and a 30% regimentation score was observed at the 3‐month mark [8].

### 3.4. Patient Satisfaction and Postoperative Complications

Two studies assessed patient satisfaction using a visual analog scale [8, 31], and three studies used a patient satisfaction questionnaire [11, 16, 32]. All of these studies showed that the use of vitamin C for gingival depigmentation led to increased patient satisfaction. Most studies reported normal healing with no major adverse outcomes; one study reported increased volume, altered texture, and changed color 1 day after the procedure for all study samples [33]. One patient reported mild itching after the first visit in one study [11], while another study observed mild pain on the first day, which gradually decreased over the following days [9]. Table 4 provides a summary of the key clinical findings and details on patient satisfaction.

**Table 4:** Summary of clinical findings.

Treatment options	Clinical outcomes
• Mesotherapy (injection): fastest, strongest depigmentation • Microneedling + vitamin C: minimally invasive, moderate speed • Topical vitamin C gel (10%): least invasive, gradual results	• 100% of studies reported pigmentation reduction • Mesotherapy showed fastest improvement • Histologic decrease in melanocyte count (one study) • Repigmentation (~30%) reported in one study only

**Patient experience**	

• High satisfaction across all methods • Reasons: minimally invasive, noticeable esthetic improvement • Adverse effects mild and temporary (itching, slight pain, and swelling)	

**Clinical takeaways**	

• Vitamin C is a minimally invasive, effective depigmentation approach • Mechanism: melanin suppression and improved tissue turnover • Best selection: rapid → mesotherapy; needle‐free → microneedling; maintenance → topical gel • Future need: standardized protocols, optimized dosing, and longer follow‐up	

### 3.5. Quality Assessment and Risk of Bias

**Figure 2:**
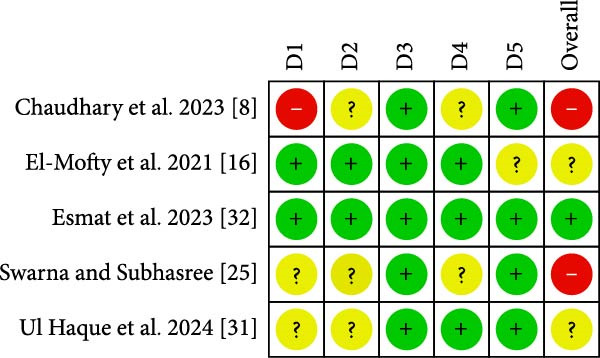
Risk of bias for RCT using RoB2: the revised Cochrane risk of bias tool for randomized clinical trial. D1: bias due to the randomization process, D2: bias due to deviation from intended intervention, D3: bias due to missing outcome data, D4: bias due to measurement of outcomes, and D5: bias due to selection of the reported result.

The authors of this study undertook detailed assessments of each article to evaluate the quality of the analysis pertinent to the research methodology. The JBI critical appraisal tools for case series studies indicated that two studies were of good quality [11, 33], while one was rated as poor quality according to the scale [9]. Table 5 contains comprehensive details about the three domains. The revised Cochrane risk of bias tool for randomized clinical trials, RoB 2, identified one study with a low risk of bias [32], two studies with some concerns [16, 31], and two studies with a high risk of bias [8, 25]. The analysis results are summarized in Figure 2.

**Table 5:** JBI critical appraisal for case series studies.

Author, year	Mostafa et al. (2023) [33]	Dawar et al. (2022) [11]	Lavanya et al. (2024) [9]
Were there clear criteria for inclusion in the case series?	Yes	Yes	No
Was the condition measured in a standard, reliable way for all participants included in the case series?	Yes	Yes	Yes
Were valid methods used for identification of the condition for all participants included in the case series?	Yes	Yes	Yes
Did the case series have consecutive inclusion of participants?	No	No	No
Did the case series have complete inclusion of participants?	Yes	Yes	Yes
Was there clear reporting of the demographics of the participants in the study?	Yes	No	No
Was there clear reporting of clinical information of the participants?	Yes	Yes	Yes
Were the outcomes or follow‐up results of cases clearly reported?	Yes	Yes	Yes
Was there clear reporting of the presenting site(s)/clinic(s) demographic information?	Yes	Yes	No
Was statistical analysis appropriate?	Yes	Yes	No
Overall appraisal	9/10	8/10	5/10

## 4. Discussion

Although vitamin C is commonly used in dermatology as a depigmentation agent, and various studies have highlighted its efficacy in reducing pigmentation caused by UV exposure [23], its use for gingival depigmentation remains underexplored. Therefore, this systematic review evaluated the current evidence on the effect of vitamin C on gingival depigmentation using various application techniques, such as mesotherapy, microneedling, and topical gel. All studies showed favorable outcomes for pigmentation reduction, with improved DOPI and GPI scores, minimal adverse effects, and high patient satisfaction.

Vitamin C is crucial for maintaining both overall and dental health, particularly because of its antioxidant properties and its involvement in collagen synthesis and wound healing. In dermatology, topical vitamin C is commonly used to treat hyperpigmentation by inhibiting tyrosinase, a key enzyme involved in melanin production. Similar effects have been observed in the oral cavity, in which vitamin C has been shown to reduce gingival pigmentation. These findings are consistent with dermatological literature, which has confirmed the efficacy of vitamin C in treating skin hyperpigmentation, suggesting that it may act similarly on melanin‐producing cells in the gingiva. However, unlike the skin, the oral environment presents unique challenges, such as constant moisture, enzymatic activity, and mucosal permeability, which may influence the treatment’s effectiveness and duration. These parallels between skin and gingival responses position vitamin C as a practical and minimally invasive alternative to conventional depigmentation techniques in dentistry [34].

The DOPI and GPI are the tools of choice in most studies, and such preference can be attributed to their relevance in clinical and research settings. The DOPI is a widely recognized clinical tool that allows for standardized, objective evaluation of gingival pigmentation intensity and distribution [4]. Its graded scoring system, typically 0–3, enhances reliability in assessing pre‐ and posttreatment pigmentation changes, particularly in esthetic or depigmentation procedures [4]. In contrast, GPI is based primarily on subjective judgment and evaluates only the color of a specific area without accounting for the spread or intensity of pigmentation, which may limit its sensitivity and reproducibility [35]. Moreover, studies have demonstrated that DOPI exhibits higher inter‐ and intraobserver reliability owing to its more structured approach, whereas GPI is more susceptible to examiner bias [23, 24].

A 10% concentration of ascorbic acid demonstrated significant efficacy in suppressing melanogenesis, particularly in the context of UV–induced pigmentation, as evidenced in clinical dermatological trials [36]. In this review, one study employed a topical formulation containing 10% ascorbic acid combined with stabilizing agents such as propylene glycol and HPMC to enhance dermal penetration and formulation integrity [16]. These excipients increase viscosity and retention on mucosal surfaces, potentially enhancing local bioavailability. Despite these advances, the inherent chemical instability of ascorbic acid, owing to its oxidation into dehydroascorbic acid and eventual conversion into biologically inactive 2,3‐diketo‐L‐gulonic acid, limits its therapeutic longevity [8]. To address this limitation, chemically modified derivatives such as 3‐O‐ethyl‐L‐ascorbic acid have been introduced to improve oxidative stability and tissue permeability [9, 32]. Although these approaches have been validated for dermatological applications, translational research focusing on oral mucosal pharmacokinetics remains limited. Further studies are necessary to determine the optimal concentration and delivery vehicles suitable for sustained gingival depigmentation.

The studies under review here employed diverse techniques for the application of ascorbic acid, reflecting both clinical versatility and methodological heterogeneity in gingival depigmentation therapy. Two main approaches were identified: mesotherapy and microneedling with topical vitamin C, each offering unique mechanisms and advantages.

The most used method was intramucosal injection (mesotherapy), which was featured in six of the eight trials under discussion [8, 9, 11, 16, 31, 32]. This approach involves the subepithelial administration of vitamin C using a fine‐gauge syringe, enabling targeted delivery to the pigmented gingival tissues. Localized deposition prolongs the retention of the active compound and enhances its diffusion into deeper layers, which may explain the superior and faster clinical outcomes observed than those resulting from topical application [13]. Mesotherapy is believed to enhance the effectiveness of depigmentation agents by creating microchannels that facilitate diffusion, while its shallow injection depth ensures that the vitamin C solution remains longer in the area being treated, promoting sustained local activity [37, 38]. This technique therefore combines precision targeting with prolonged therapeutic effect, resulting in noticeable pigmentation reduction and patient satisfaction with minimal adverse effects. Moreover, mesotherapy has been found to significantly improve drug delivery through microchannels that enhance the penetration of topical treatments such as serums containing vitamins or growth factors; this increased absorption can significantly boost the effectiveness of these treatments [38].

In contrast, microneedling offers a minimally invasive alternative that enhances the topical absorption of ascorbic acid. Two studies [25, 33] reported microneedling using a Dermapen device, which created controlled microperforations in the gingival epithelium, followed by applying ascorbic acid powder. This procedure increases tissue permeability and facilitates deeper vitamin C penetration without injections, representing a favorable option for patients seeking noninvasive esthetic correction. Although microneedling produced significant improvements in gingival pigmentation, outcomes were generally slower to manifest than those achieved through mesotherapy, likely due to differences in tissue diffusion and concentration delivery. Treatment parameters, including needle depth, frequency, and vitamin C formulation, also varied among studies, highlighting the need for standardized protocols to optimize outcomes.

Based on the treatment schedules reported in the studies being discussed, both the duration and frequency of vitamin C application varied depending on the delivery technique. In most mesotherapy studies, subepithelial administration of vitamin was done using a fine‐gauge syringe once weekly for three to four sessions, allowing localized retention of the agent and promoting deeper diffusion within gingival tissues [8, 9, 11, 16, 31, 32]. Microneedling combined with topical vitamin C was typically performed in two to three sessions at weekly intervals, using a Dermapen‐type device to create controlled microperforations followed by the immediate application of ascorbic acid powder or solution [25, 33]. In contrast, topical‐only regimens generally involved daily application of 10% ascorbic acid gel for 8 to 12 weeks, producing gradual depigmentation but requiring longer treatment time and strong patient compliance [16]. Follow‐up durations ranged from 2 weeks to 6 months across studies, with limited evidence regarding long‐term stability [8, 11, 16, 31–33].

From these data, a practical clinical approach may involve initiating therapy with mesotherapy once per week for 3 to 4 weeks when injections are acceptable and rapid esthetic improvement is desired. For patients preferring noninvasive options, microneedling with topical vitamin C performed weekly for two to three sessions can serve as an effective alternative. After either active intervention, daily use of 10% vitamin C gel for 8 to 12 weeks may help maintain depigmentation and extend esthetic outcomes. Regardless of the technique used, clinicians should emphasize plaque control, discourage smoking, and tailor retreatment schedules to individual response until standardized, long‐term protocols are established [8, 11, 16, 31–33].

Focusing specifically on topical vitamin C, outcomes tend to be less pronounced and slower to appear than those resulting from injection‐based or energy‐driven techniques. In the only direct comparison available, mesotherapy achieved faster and more effective pigmentation reduction than topical gel application [16]. Although topical vitamin C alone may not match the efficacy of laser or surgical depigmentation [2, 13–15, 35], it remains valuable for patients seeking noninvasive options and can serve as an adjunct to penetration‐enhancing procedures such as microneedling [25, 33] or as maintenance therapy following definitive treatment.

When evaluating the efficacy of vitamin C–based approaches, it is essential to consider them in relation to established depigmentation techniques. Conventional methods—such as scalpel surgery, gingival abrasion, electrosurgery, cryosurgery, and a variety of laser systems (CO_2_, diode, Er:YAG, and Nd:YAG)—have long been employed to remove gingival pigmentation and achieve esthetic improvement [2, 13–15, 35]. These techniques generally produce reliable pigment elimination; however, they are not minimally invasive, frequently require local anesthesia, and are often accompanied by postoperative discomfort, bleeding, or delayed healing. Additionally, the cost of laser equipment and the level of operator expertise needed may restrict their routine clinical use.

In contrast, vitamin C–based treatments, including mesotherapy, microneedling with topical vitamin C, and direct topical application, provide conservative alternatives that minimize tissue trauma while achieving satisfactory depigmentation [13, 28–30, 32, 33]. Current evidence indicates that these techniques can yield comparable esthetic results to surgical and laser procedures, with fewer side effects and high patient satisfaction.

Beyond comparative efficacy, dosage and formulation differences also influence treatment outcomes. Across the studies under review, the dose and concentration of vitamin C varied substantially with the delivery method. For intramucosal mesotherapy, ~0.1 mL of injectable vitamin C solution (100–1000 mg/mL) was administered per site, while topical regimens used a 10% ascorbic acid gel applied daily for up to 3 months [8, 9, 11, 16, 25, 31–33]. These findings indicate that injectable formulations deliver higher localized concentrations over a shorter duration, whereas topical gels rely on sustained exposure for cumulative depigmenting effects.

Variations in delivery routes, including, injectable, topical, and combination therapies, highlight the absence of a standardized clinical protocol. Despite these methodological differences, all approaches demonstrated measurable efficacy, with injectable forms producing faster results and microneedling‐assisted topical therapy showing strong potential. Patients frequently favored vitamin C–based treatments not only for their minimally invasive nature but also for their affordability compared with laser or surgical depigmentation techniques, and these factors enhance their appeal in resource‐limited settings.

Across these studies, ascorbic acid was applied in different concentrations and formulations, including powder form, which was used in two studies [25, 33]. Such inconsistency makes it difficult to determine the optimal concentration for achieving stable, long‐term outcomes. Moreover, substantial heterogeneity among the studies—arising from differences in application technique, formulation type, dosing frequency, and evaluation methods—limits direct comparison of results. The reliance on clinical observation and photographic documentation further emphasizes the need for standardized, quantitative, and reproducible protocols to improve consistency and reliability in future investigations.

This systematic review offers a comprehensive synthesis of the current literature on vitamin C–based depigmentation therapies, covering injectable, topical, and microneedling‐assisted techniques. The inclusion of multiple study designs and adherence to PRISMA guidelines strengthen the validity of its findings. However, the evidence base remains limited by the small number of clinical trials, variability in dosage and delivery methods, and the short follow‐up durations of most studies. The absence of quantitative meta‐analysis and the reliance on subjective outcome measures, such as photographic assessment, further restrict generalizability.

## 5. Conclusions

Current evidence supports the short‐term efficacy of ascorbic acid as a minimally invasive treatment for gingival hyperpigmentation. However, substantial variability in application techniques, concentrations, and evaluation methods limits the ability to draw definitive conclusions. Available studies consistently report favorable clinical outcomes, minimal adverse effects, and high patient satisfaction, particularly when compared with surgical and laser‐based approaches. To strengthen the evidence base and establish clear clinical guidelines, future investigations should prioritize well‐designed randomized controlled trials that use standardized protocols, extended follow‐up periods, and objective outcome measures.

## Ethics Statement

No ethical approval was required to complete this systematic review.

## Disclosure

All authors reviewed the results and approved the final version of the manuscript.

## Conflicts of Interest

The authors declare no conflicts of interest.

## Author Contributions

Study conception and design: **Bushra Aljahdali**. Data collection: **Bushra Aljahdali** and **Rana Dahlan**. Analysis and interpretation of results: **Bushra Aljahdali** and **Rana Dahlan**. Draft manuscript: **Bushra Aljahdali** and **Rana Dahlan**.

## Funding

The authors did not receive any financial support for this systematic review. Open Access publishing facilitated by the Deanship of Scientific Research (DSR) at King Abdulaziz University, as part of the Wiley ‐ King Abdulaziz University agreement.

## Data Availability

This study is a systematic review of previously published literature. All data extracted and analyzed are publicly available from the original studies cited in the manuscript. No new primary data were generated.
